# Functional and structural characterization of osteocytic MLO-Y4 cell proteins encoded by genes differentially expressed in response to mechanical signals in vitro

**DOI:** 10.1038/s41598-018-25113-4

**Published:** 2018-04-30

**Authors:** Fanchi Meng, Graeme F. Murray, Lukasz Kurgan, Henry J. Donahue

**Affiliations:** 1 Department of Electrical and Computer Engineering, University of Alberta, Edmonton, Canada; 2 Bone Engineering, Science and Technology (BEST) Laboratory, Department of Biomedical Engineering, Virginia Commonwealth University, Richmond, Virginia United States of America; 3 Department of Computer Science, Virginia Commonwealth University, Richmond, Virginia United States of America

## Abstract

The anabolic response of bone to mechanical load is partially the result of osteocyte response to fluid flow-induced shear stress. Understanding signaling pathways activated in osteocytes exposed to fluid flow could identify novel signaling pathways involved in the response of bone to mechanical load. Bioinformatics allows for a unique perspective and provides key first steps in understanding these signaling pathways. We examined proteins encoded by genes differentially expressed in response to fluid flow in murine osteocytic MLO-Y4 cells. We considered structural and functional characteristics including putative intrinsic disorder, evolutionary conservation, interconnectedness in protein-protein interaction networks, and cellular localization. Our analysis suggests that proteins encoded by fluid flow activated genes have lower than expected conservation, are depleted in intrinsic disorder, maintain typical levels of connectivity for the murine proteome, and are found in the cytoplasm and extracellular space. Pathway analyses reveal that these proteins are associated with cellular response to stress, chemokine and cytokine activity, enzyme binding, and osteoclast differentiation. The lower than expected disorder of proteins encoded by flow activated genes suggests they are relatively specialized.

## Introduction

Mechanical fluctuations in the extracellular environment, such as interstitial fluid flow-induced shear stress, induce cell perturbations including membrane deformations, cytoskeletal restructuring, conformational changes in transmembrane proteins, changes in the glycol calyx and movement of cilia. These cell responses to mechanical signals are translated throughout the cell and can result in alterations in gene expression across many tissues^1^.

In bone, osteocytes are well positioned to detect mechanical signals from loading and communicate them to downstream effector cells including osteoblasts, osteoclasts, and bone lining cells^2^. However, the signal transduction pathways involved in this process are only partially understood. A better understanding of signaling pathways activated by mechanical signals *in vitro* may lead to a better understanding of how bone adapts to load *in vivo*.

We have previously used gene microarrays, proteomics and RNA sequencing analysis (RNA-Seq) to identify increases in inflammatory C-X-C motif chemokines, including Ccl2, and the HIF-1α, IL-17, and AMPK signaling pathways induced by fluid flow^3,4^. However, these studies were limited as they did not examine functional and structural characteristics of proteins encoded by the corresponding differentially expressed genes and have focused only on genes with the highest signal. To address this we analyzed previously obtained RNA-seq data and gene micro array data^3,4^ using several high-throughput computational methods to quantify evolutionary conservation, interconnectedness, and putative intrinsic disorder of the proteins encoded by all differentially expressed genes. We also functionally characterized these proteins using pathway analysis and gene ontology. We analyzed which of these characteristics are unique to proteins encoded by genes affected by fluid flow by comparing them to a generic set of proteins from the mouse proteome.

Conservation quantifies the amount of evolutionary variation in a protein sequence across species. When measured at the protein level it reflects the abundance of functionally and structurally important residues that are typically retained across species^5–7^. Interconnectedness measures the number of interactions between proteins in protein-protein interaction (PPI) networks, and can predict, along with conservation, the extent to which this protein is essential^8,9^ and central to cellular processes^10^. Intrinsic disorder reflects the degree to which a protein lacks an ordered 3D structure under physiologic conditions^11,12^. While the levels of intrinsic disorder vary from protein to protein, disorder itself is ubiquitous. Recent estimates show that about 19% of amino acid residues are disordered in Eukaryotes^13^ and between 6% and 17% of proteins encoded by various genomes are entirely disordered^14^. Intrinsic disorder is implicated in a diverse range of cellular functions including transcription^15,16^, translation^17^, protein-protein interactions^18,19^, protein-RNA interactions^20,21^ and cell signaling^19,22,23^, to name a few. Functional characteristics that are specific to disorder include one-to-many binding, where one disordered region can interact with several structurally diverse partners; ability to fold upon binding; high accessibility to and regulation by post-translational modifications; and ability to implement cascade interactions^24^. At the same time, a high level of disorder is a substantial obstacle to solving protein structure^25–27^ and to performing rational drug design that relies on knowledge of protein structure^28–30^.

Gene ontology (GO) is a bioinformatics framework that defines biological processes, molecular functions and subcellular location of genes and proteins^31,32^. These annotations allow for the computational identification of functions and locations that are characteristic of a given group of genes or proteins. In a similar manner, pathway and network analysis can compare differentially expressed proteins and genes to known pathways to identify possible downstream effects.

We hypothesized that examining protein conservation, interconnectedness, intrinsic disorder, as well as pathway analysis, would provide insights to the function of osteocytic proteins encoded by genes differentially expressed in response to fluid flow-induced shear stress. This is the first study to examine evolutionary conservation, interconnectedness and putative intrinsic disorder in the context of mechanobiology.

## Results

**Figure 1:**
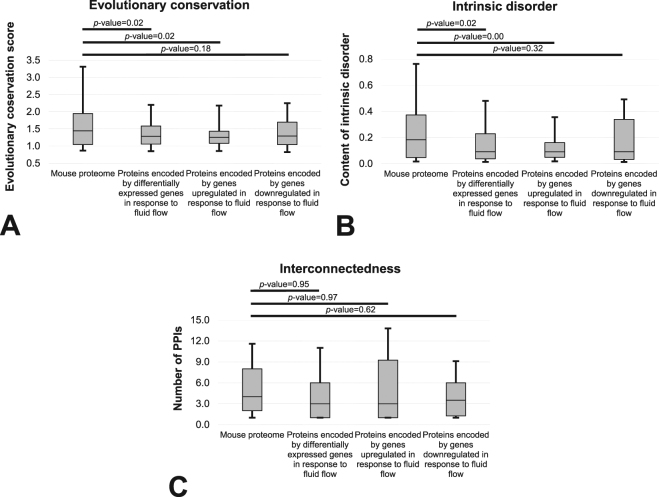
Comparison of the distrubutions of the evolutionary conservation quantified with the relative entropy scores (Panel A), content of the intrinsic disorder (Panel B) and the interconnectedness measured by the number of PPIs (Panel C) between the mouse proteins encoded by genes that are differentially expressed in response to fluid flow and a generic set of mouse proteins. The differentially expressed gene sets are also subdivided into upregulated and downregulated proteins. Distributions are represented with the box and whisker diagrams where the first quartile, second quartile (median) and third quartile are represented by the box and the whiskers correspond to the 10^th^ and 90^th^ centiles. Horizontal bars at the top of the figure summarize results of the analysis of statistical significance of the differences between the distribution for the two corresponding datasets. The significance was quantified with the two sample K-S test and the corresponding *p*-values are shown above the bars.

We comprehensively characterized several major structural and functional characteristics of osteocytic proteins encoded by genes differentially expressed due to exposure to fluid flow-induced shear stress. Figure 1 summarizes results focusing on evolutionary conservation, content of putative intrinsic disorder and interconnectedness. We compared the entire complement of proteins encoded by differentially expressed genes as well as proteins encoded by genes upregulated and downregulated against a generic set of murine proteins. Figure 1A reveals that proteins that correspond to the differentially expressed genes, and in particular proteins encoded by genes that are upregulated, have significantly lower than expected evolutionary conservation (*p*-value < 0.05). Proteins encoded by genes that are downregulated are also characterized by lower levels of conservation, however, the difference did not reach statistical significance when *p*-value considered significant was set at 0.05 (*p*-value = 0.18). Figure 1B reveals similar observations for the content of intrinsic disorder. That is, proteins encoded by differentially expressed and upregulated genes have significantly lower than expected disorder content (*p*-value < 0.05). Proteins encoded by downregulated genes also have lower disorder content, however, the difference did not reach statistical significance when p value considered significant was set at 0.05 (*p*-value = 0.32). The median disorder content across proteins encoded by differentially regulated genes equals 0.09 compared to the twice larger (0.18) content that is typical for murine proteins^13^. On the other hand, the degree of interconnectedness is not significantly different. Proteins encoded by differentially expressed genes, including both upregulated and downregulated subsets, have similar levels of PPIs (*p*-values > 0.6) compared to an overall set of murine proteins. The average number of PPIs in mouse listed in the metha resource equals 5.97^33^ and is, as expected, virtually identical to the number that we report for our randomly chosen subset of murine proteins (6.03), and only slightly higher than for the proteins encoded by differentially expressed genes (5.63).

**Figure 2:**
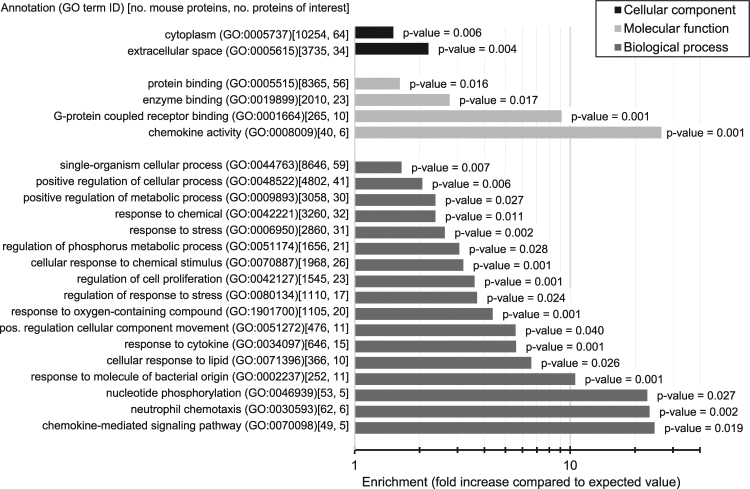
Summary of GO terms significantly enriched (*p*-value < 0.05) in the mouse proteins encoded by genes differentially expressed in response to fluid flow. The analysis was peformed using the PANTHER system^67^ separately for each of the three classes of GO terms: molecular functions, biological processes and cellular component (subcellular location). Horizontal bars show the value of enrichment, defined as the fold increase when compared to the expected value measured on the mouse proteome, and the corresponding *p*-values are given next to the bars. The number of each GO term occurence in the mouse proteome and among the proteins encoded by differentially expressed genes is given inside the square brackets. We only consider GO terms that occur at least five time and for which the enrichment is greater than 150% and *p*-value < 0.05.

For GO analyses, we collected the most frequent term for each path in the GO hierarchy to reduce redundancy between the terms and to include the most frequent terms. Figure 2 depicts a list of subcellular locations (black bars), molecular functions (light gray bars) and biological processes (dark gray bars) that are significantly enriched in the proteins encoded by differentially expressed genes (*p*-value < 0.05 and at least 50% increase compared to the expected value to ensure that magnitude of the difference is substantial). Our genes of interest are primarily located in the cytoplasm and extracellular space. The GO terms that have high enrichment and high counts (second number inside square brackets next to the GO term name in Fig. 2) suggests that they are involved in chemokine (GO:0008009 and 0070098) and cytokine (GO:0034097) activities, response and regulation of response to stress (GO:0006950 and 0080134), and cellular response to chemical stimulus and oxygen-containing compound (GO:0070887 and 1901700). Annotations of molecular functions (light gray bars) also suggest that they take part in enzyme and G-protein coupled receptor binding.

**Figure 3:**
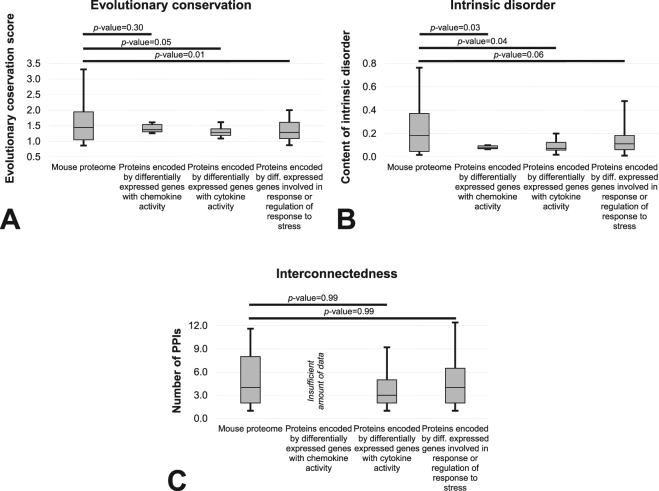
Comparison of the distrubutions of the evolutionary conservation quantified with the relative entropy scores (Panel A), content of the intrinsic disorder (Panel B) and the interconnectedness measured by the number of PPIs (Panel C) between functionally clustered subsets of the mouse proteins coded by genes that are differentially expressed in response to fluid flow (proteins of interest (POI)) and a generic set of mouse proteins. We considered three clusters that correspond to the proteins with chemokine activity (GO:0008009; 6 proteins), cytokine activity (GO:0034097; 15 proteins) and to those that are involved in the response of regulation of response to stress (GO:0006950 and 0080134; 35 proteins). Five of them were identified across the three clusters and additional eight are in common between the latter two clusters. Only three proteins encoded by differentially expressed genes with the cytokine activity had the PPI information and thus the analysis was not performed due to the low sample size. Distributions are represented with the box and whisker diagrams where the first quartile, second quartile (median) and third quartile are represented by the box and the whiskers correspond to the 10^th^ and 90^th^ centiles. Horizontal bars at the top of the figure summarize results of the analysis of statistical significance of the differences between the distribution for the two corresponding datasets. The significance was quantified with the two sample K-S test and the corresponding *p*-values are shown above the bars.

We further focused on several key functions related to chemokine and cytokine processes and response and regulation of response to stress. We visualized the distributions of the evolutionary conservation, putative intrinsic disorder and interconnectedness for these proteins encoded by differentially expressed genes and compared them to a generic set of murine proteins (Fig. 3). Consistent with the results in Fig. 1, conservation (Fig. 3A) and amount of intrinsic disorder (Fig. 3B) are significantly lower than expected in these three sets of proteins that correspond to the differentially expressed genes (*p*-value < 0.05, except for the difference in conservation for proteins with chemokine activity where *p*-value = 0.3). Moreover, the numbers of PPIs are similar to a generic murine protein (*p*-value = 0.99).

**Figure 4:**
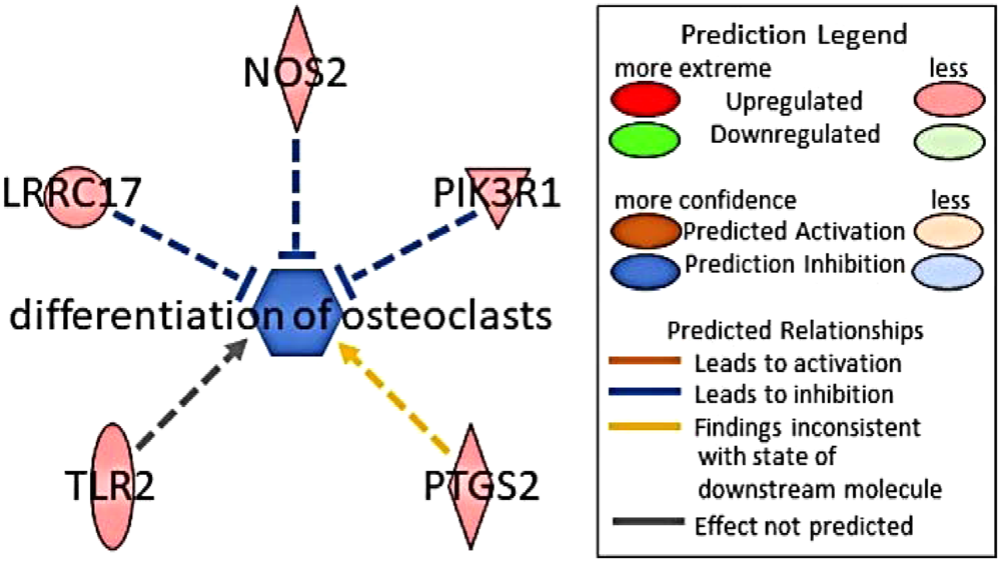
IPA predicted inhibition of the differentiation of osteoclasts with an activation Z score of −1.07 with a *p*-value < 0.001.

Ingenuity Pathway Analysis constructs causal networks from individual relationships detailed in curated literature^34^. Using these networks, downstream effects can be predicted based on our data. Casual network analysis of our RNA-seq data found a fluid flow-induced upregulation of proteins encoded by differentially expressed genes that are associated with the downregulation of osteoclast differentiation (Fig. 4). Shown in Fig. 4 are all the proteins that correspond to the differentially expressed genes in our dataset expected to influence osteoclast differentiation. NOS2, LPRC17 and PLK3R1 that are associated with inhibition of osteoclast differentiation were moderately upregulated; PTGS2 was also upregulated but would be predicted to increase osteoclast differentiation while TLR2 may effect osteoclast differentiation, IPA’s database did not have enough power to generate a prediction. NOS2, TLR2, and PTGS2 all play a role in the regulation of the inflammatory response which is consistent with our gene ontology analysis which found increase of cytokines and chemokines.

## Discussion

Our results suggest that proteins encoded by genes that are differentially expressed in response to fluid flow have lower than expected levels of sequence conservation and putative intrinsic disorder while maintaining typical levels of PPIs. Given their relatively low conservation, they are likely to carry out more specialized (non-essential) functions compared to the functions of proteins encoded by essential genes that have older evolutionary origins and are more conserved^34–36^. Proteins encoded by these essential genes are typically involved in basic cellular functions including gene expression, metabolism, morphogenesis, cell division, proliferation and differentiation, DNA replication, repair and transcription, and embryonic development and they are essential to the survival of the organism^34^. At the same time, the levels of interconnectedness of the proteins encoded by differentially expressed genes that are comparable to a typical mouse protein suggests that they are functionally important even though they may not be essential. The significant depletion in the putative intrinsic disorder suggests that they are highly structured and substantially more structured than a typical murine protein. The main implication of this observation is that structures of the proteins encoded by differentially expressed genes should be relatively easy to produce^25,37^. These structures can be used to decipher molecular level details of their functions^38^ and they are necessary for virtual screening and design of novel therapeutics^39,40^. Taken together, our analyses suggest that genes involved in mechanotransduction are non-essential, functionally important, and suitable for structure-based rational drug design. We note that the above observations rely on the putative intrinsic disorder and an incomplete PPI network, both of which may adversely affect accuracy of our analysis. However, we designed the analysis to reduce these effects. We employed a consensus of five predictors of disorder to minimize the prediction error and we used a recently released database that collects PPIs from a comprehensive set of five sources to provide the currently most complete network. Additionally, there are limitations to our fluid flow model that are shared by several *in vitro* models. These include analyses of only a single bone cell type, the two dimensional nature of our fluid flow apparatus and the fact that the cells are not in contact with a physiologically relevant extracellular matrix.

In this study we used IPA to assess RNA-seq data. We opted to assess RNA-seq data, rather than microarray data, because we previously showed that RNA-Seq yielded greater magnitude fold-changes in expression level than did microarrays, resulting in a broader overall dynamic range^3^. Furthermore, RNA-seq, compared to microarray was capable of detecting approximately 3 times the gene products of previously utilized microarrays. Our IPA analyses predicted fluid flow-induced changes in cytokines consistent with a down regulation of osteoclastogenesis, which one would expect as a result of exposure to anabolic mechanical signals. Additionally, consistent with our results, osteocytes have been shown to downregulate osteoclast differentiation through effects on TGF-β, an upstream inducer of IL-17, a factor that increases osteoclastogenesis^41–43^. On the other hand, our previous work showed fluid flow-induced upregulation of pathways dependent on IL-17^4^ and another study showed that mechanical load upregulates TGF-β in mesenchymal stem cells^44^. These findings emphasize the complexity of the bone microenvironment and suggest the effects of cytokines on bone are highly context dependent. Ultimately, cytokines can have either anabolic or catabolic effects on bone which has complicated efforts at treatment of metabolic bone disease^45^. This complexity is illustrated by IL-17 which can be upregulated in both catabolic and anabolic states. Phase II trials of IL-17 inhibitors such as secukinumab for rheumatoid arthritis have yielded disappointing results^46^. Thus, there is a need for further research in this area to characterize the bone microenvironment an how it is affected by mechanical load, especially as regards the pleiotropic effect of cytokines in bone. The complexity of microenvironment also suggest the therapeutic target potential of mechanotransduction pathways because we have shown them to be less essential while still functionally important when compared to the mouse proteome.

In summary, this is the first study to quantify the structural and functional characteristics such as intrinsic disorder, evolutionary conservation, and interconnectedness of the proteins encoded by differentially expressed genes involved in mechanotransduction. These results suggest that the osteocytic signaling pathways activated by fluid flow are non-essential and populated by highly structured proteins. Furthermore, extracellular inflammatory mediators are involved in the downregulation of osteoclast differentiation. The well-defined tertiary structure of proteins encoded by differentially expressed genes in this pathway suggests that protein modeling could be successful in developing a better understanding of the signaling molecules involved in the response of bone to mechanical load.

## Methods

### Collection of experimental data

In the original study^3^, murine osteocytic MLO-Y4 cells were cultured in normal growth medium (α-MEM [Invitrogen, Grand Island, NY] with 2.5% calf serum [Hyclone, Logan, UT], 2.5% fetal bovine serum [Lonza, Walkersville, MD], 1% Penicillin/Streptomycin) throughout all portions of the experiment. Cells were seeded 48 h prior to fluid flow on 75 × 38 × 1 mm glass slides coated with 300 μg/ml Type I Collagen (BD Biosciences, Bedford, MA) for 1 h and washed. Cell seeding density was 1.35 × 10^4^ cells/cm^2^ so that upon flow exposure, cells were roughly 60% confluent and interconnected by dendritic processes. Cells were then exposed to 2 hours of sinusoidally oscillating fluid flow in parallel plate flow chambers, inducing 1 Pa (10 dynes/cm^2^) shear stress at 1 Hz. Paired sham controls were maintained in identical, static chambers. Triplicates of both flowed and static cells were collected following post-incubation in fresh medium for 2 hours.

Total RNA was isolated using Qiagen RNeasy Mini Kits, deep sequenced using Illumina HiSeq 2500, and processed computationally to identify genes that are differentially expressed between the samples exposed to the fluid flow and the controls^4^. Similar and complementary analysis was performed using DNA microarrays to identify further gene products that are characterized by differential abundance^3^. In both cases only the genes characterized by significant differences (*p*-values < 0.05) were selected. These data are available in the Gene Expression Omnibus with accession number GSE70667 for the RNA-Seq and GSE42874 for the microarrays.

### Dataset

Our prior analyses revealed two sets of 55 differentially expressed genes based on the RNA-Seq and microarray experiments. After removing duplicates of the 6 genes that were in common between the two sets, we mapped 99 genes to their UniGene identifiers. The remaining 5 genes could not be mapped to these identifiers. Next, we searched the UniProt resource^47,48^ to map the UniGene identifiers to the corresponding mouse proteins. Once we removed duplicate proteins and protein fragments, we extracted 103 proteins encoded by the 99 differentially expressed genes that we were able to map into unique UniProt accession numbers. Among the 103 proteins encoded by the differentially expressed genes, 45 and 58 that are significantly upregulated or downregulated by fluid flow, respectively.

### Analysis

The 103 proteins encoded by differentially expressed genes in response to fluid flow were subjected to a comprehensive bioinformatics analysis. We contrasted their structural and functional characteristics against the reference mouse proteome collected from UniProt (C57BL/6 J strain, proteome ID: UP000000589). Since the amount of intrinsic disorder in eukaryotic organisms was shown to depend on the protein length^49^, we randomly selected a matching number of mouse proteins to obtain the same distribution of lengths when compared to our 103 proteins encoded by differentially expressed genes. This ensures that our comparison of intrinsic disorder accommodates sequence-length bias; the same correction was made in several recent studies^50,51^. Equalization of sample size (103 proteins encoded by differentially expressed genes plus 103 proteins sampled from the reference proteome) also ensures that these data are suitable for robust statistical analysis. This is in contrast to using the whole mouse proteome of over 50 thousand proteins, which would result in over two orders of magnitude difference in sample size relative to proteins differentially expressed in response to fluid flow. The two sets have virtually identical distributions of protein length; *p*-value = 1 based on the two-sample Kolmogorov-Smirnov test. The difference in their median chain length, which is 412 and 416 for the random set of mouse proteins and our proteins encoded by differentially expressed genes, respectively, is not significant; *p*-value = 0.97 based on the Mann-Whitney test.

We estimated evolutionary conservation from multiple alignment profiles generated with HHblits against the *UniProt-20* database^52^. These profiles were used to compute relative entropy from each amino acid^53,54^. An average per-amino acid conservation was used to quantify conservation for each proteins encoded by differentially expressed genes. We collected the mouse PPI network from the *mentha* resource that integrates data from five manually curated source databases^33^. With that integrative approach, the mentha resource offers arguably the most complete currently available PPI network in mouse. The degree of interconnectedness was quantified by its number of PPIs. We annotated putative intrinsic disorder using a majority-vote based consensus of five predictors: three versions of the Espritz method^55^ and two versions of the IUPred method^56^; These methods are characterized by complementary designs and were empirically demonstrated to offer strong predictive quality for the prediction of intrinsic disorder^57–59^. We used the consensus to minimize the prediction error, which is in line with an observation that this results in a better predictive quality when compared to the use of individual predictors^60,61^. Such consensuses are also implemented in the current databases of putative disorder: MobiDB^62,63^ and D^2^P^2 ^^64^, and were utilized in many other studies^13,17,21,50,51,65,66^. We quantified amount of disorder with the disorder content, which is defined as a fraction of disordered amino acids in a given sequence. We measured statistical significance of differences in distributions of conservation, disorder and interconnectedness values between our proteins of interest and the mouse proteome using a two-sample Kolmogorov-Smirnov test (K-S test) and assumed that a given difference is significant at *p*-values < 0.05.

We performed functional analysis using GO with the PANTHER system^67^. PANTHER performs enrichment analysis of GO terms associated with our proteins encoded by differentially expressed genes against the expected frequency of these terms in the mouse proteome^68^. We executed this analysis separately for each of the three classes of GO terms: molecular functions, biological processes and cellular component (subcellular location). The pathway and causal network analyses was completed using the Ingenuity Pathway Analysis (IPA) tool^69^.
